# A Targeted Mass Spectrometric Assay for Reliable Sensitive Hepcidin Quantification

**DOI:** 10.1038/s41598-019-43756-9

**Published:** 2019-05-13

**Authors:** Ahmed Moghieb, Lia Tesfay, Song Nie, Marina Gritsenko, Thomas L. Fillmore, Jon M. Jacobs, Richard D. Smith, Frank M. Torti, Suzy V. Torti, Tujin Shi, Charles Ansong

**Affiliations:** 1 Biological Science Division, Pacific Northwest National Laboratory, Richland, WA USA; 2 Departments of Molecular Biology and Biophysics, University of Connecticut, Farmington, CT USA; 3 Department of Medicine, UConn Health Center, Farmington, CT USA

**Keywords:** Assay systems, Mass spectrometry

## Abstract

Hepcidin, a cysteine-rich peptide hormone, secreted mainly by the liver, plays a central role in iron metabolism regulation. Emerging evidence suggests that disordered iron metabolism is a risk factor for various types of diseases including cancers. However, it remains challenging to apply current mass spectrometry (MS)-based hepcidin assays for precise quantification due to the low fragmentation efficiency of intact hepcidin as well as synthesis difficulties for the intact hepcidin standard. To address these issues we recently developed a reliable sensitive targeted MS assay for hepcidin quantification from clinical samples that uses fully alkylated rather than intact hepcidin as the internal standard. Limits of detection and quantification were determined to be <0.5 ng/mL and 1 ng/mL, respectively. Application of the alkylated hepcidin assay to 70 clinical plasma samples (42 non-cancerous and 28 ovarian cancer patient samples) enabled reliable detection of endogenous hepcidin from the plasma samples, as well as conditioned culture media. The hepcidin concentrations ranged from 0.0 to 95.6 ng/mL across non-cancerous and cancer plasma specimens. Interestingly, cancer patients were found to have significantly higher hepcidin concentrations compared to non-cancerous patients (mean: 20.6 ng/ml for cancer; 5.94 ng/ml for non-cancerous) (p value < 0.001). Our results represent the first application of the alkylated hepcidin assay to clinical samples and demonstrate that the developed assay has better sensitivity and quantification accuracy than current MS-based hepcidin assays without the challenges in synthesis of intact hepcidin standard and accurately determining its absolute amount.

## Introduction

Hepcidin is a peptide hormone derived from the liver and known to regulate systemic iron hemostasis^1,2^. The mature bioactive form of hepcidin is a 25 amino acid residue peptide (hepcidin-25). Smaller isoforms of hepcidin have been reported in serum and urine, but their functional significance is unclear^3–6^. Hepcidin dysfunction is associated with several diseases, including various cancers, sepsis and chronic kidney disease^7,8^ where in general hepcidin concentrations are increased. Thus, there is growing interest in utilizing hepcidin measurement as a more instructive marker for iron state, as well as diagnosis and management of iron metabolism disorders. Immunoassays have traditionally been utilized to measure hepcidin in biological fluids. However, present immunoassays measure total hepcidin and do not distinguish hepcidin forms (e.g., hepcidin-20, hepcidin-22, and hepcidin-25). Mass spectrometry (MS)-based assays for hepcidin detection have been reported^4,9–13^. The attractiveness of MS-based assays stems from the fact that they are able to distinguish hepcidin isoforms. Literature reports of MS-based hepcidin concentrations indicate significant variations^14^, most likely due to the lack of transferable hepcidin gold standards. Intact hepcidin has 4-disulfide linkages^15^, making consistent synthesis of intact hepcidin standard with high purity challenging, and thus degrading quantitation accuracy. To our best knowledge, the only prior report utilizing the reduced form of hepcidin-25 was by Cho *et al*.^16^ who compared the ionization efficiency of reduced and nonreduced forms in a targeted mass spectrometry assay with relatively low sensitivity (LOD 1 fmol in an un-reported matrix) and who did not apply their approach to clinical samples.

Herein we describe an improved MS-based assay that addresses the challenge(s) with intact hepcidin-25 by using a fully alkylated hepcidin-25 and the first application of the alkylated hepcidin assay to clinical samples. The use of an alkylated hepcidin-25 standard makes both synthesis of a consistent peptide product and accurate determination of hepcidin-25 straightforward. We show that the alkylated hepcidin-25 alleviates the low fragmentation efficiency of intact hepcidin-25. Additionally, we demonstrate that assays for alkylated hepcidin-25 provide improved detection (LOD) and limit of quantification (LOQ) values in plasma compared to current MS-based assays using intact hepcidin-25 as internal standards. Finally, we demonstrate the improved assay to measure endogenous hepcidin-25 in 70 clinical plasma samples (42 non-cancerous and 28 ovarian cancer patient samples) as well as in conditioned culture media.

## Experimental Procedures

### Reagents

Urea, iodoacetamide (IAA), and trichloroacetic acid (TCA) were purchased from Sigma (St. Louis, MO). Tris(2-carboxyethyl) phosphine (TCEP) was purchased from Thermo Scientific (San Jose, CA). Mass spectrometry grade solvent components, including water (H_2_O), formic acid (FA), and acetonitrile (ACN) were purchased from Sigma (St. Louis, MO).

### Hepcidin-25 standards

The synthetic fully alkylated hepcidin-25 peptide (DTHFPICIFCCGCCHRSKCGMCCKT) labeled with ^13^C/^15^N on C-terminal lysine residue (K^24^), 3259.97 g/mol and unlabeled, 3251.26 g/mol were purchased from New England Peptides (Gardner, MA). The alkylated light peptide for hepcidin-25 were estimated to be of >95% purity by HPLC and the purity of crude alkylated heavy hepcidin-25 was not known.

### Sample preparation

All plasma samples (including 70 patient plasma) were subjected to preparation workflow for precise detection and quantification of hepcidin-25 as shown in Supplementary Fig. 1. First, samples were thawed in ice and then vortex mixed. In a 1.5 mL LoBind tube, 100 µl plasma proteins were mixed with 10 µl heavy internal standard (71.03 ng/ml), and then denatured with 8 M urea and reduced with 10 mM TCEP for 2 h at 37 °C. Protein cysteine residues were alkylated with 40 mM iodoacetamide for 2 h at room temperature in the dark. 4% of trichloroacetic acid (TCA) solution (1:1 ratio) was added. Samples were then vortexed for a few seconds and centrifuged at 18,000 × g for 5 minutes to obtain a clear supernatant. The supernatant was transferred into a new LoBind tube, then diluted with 0.1% formic acid in water for solid phase extraction (SPE) cleaning using a 1 mL SPE C18 column (Phenomenex, Torrence, CA). After SPE cleaning, the samples were completely dried in a vacuum concentrator, resuspended with 40 µl 0.1% formic acid in water and then centrifuged at 18,000 × g for 3 h at 4 °C. The supernatant was transferred into liquid chromatography (LC) vials (Waters) for mass spectrometry (MS) analysis. For media, in 15 ml tube, 2 ml media was mixed with 10 µl internal hepcidin-25 standard solution (71.03 ng/ml), using the same protocol as described above for plasma samples with only one extra step of bovine serum albumin (BSA) blocking of SPE C18 columns prior to media sample cleaning.

### Generation of calibration curve

Control plasma with negligible endogenous hepcidin-25 was used as the matrix for generating calibration curve. Lyophilized high-purity light alkylated hepcidin-25 peptide was reconstituted with H_2_O/ACN (70:30, v/v), then hepcidin-25 stock solutions (16.26, 162.56 and 1625.63 ng/ml) were diluted with the same solvent. Light alkylated hepcidin-25 was spiked into the control plasma with the final concentrations of 0, 0.5, 1, 2.5, 5, 10, 25, 50, 100 and 250 ng/mL. Heavy alkylated synthetic peptide was also spiked into each sample at a final concentration of 71.03 ng/ml. The spiked-in samples were processed according to the sample preparation described above. The generated calibration curve was used for calculating the endogenous hepcidin-25 concentrations in clinical samples, alkylated heavy synthetic peptide, and for determining the LOD and LOQ values.

### Human plasma specimens

The use of human blood plasma samples was approved by the Institutional Review Boards of the University of Connecticut and Pacific Northwest National Laboratory in accordance with federal regulations. Clinical plasma samples were collected from the biorepository of UCHC with informed consent given for the use of samples in research (IRB IE-08-310-1). The control (non-cancerous) and the cancer plasma specimens were obtained from healthy people with no cancer diagnosis and female diagnosed with ovarian cancer, respectively.

### Media samples

HepG2 cells were obtained from American Type Culture Collection (ATCC) and cultured in Eagle’s Minimum Essential Medium (EMEM medium) from ATCC containing 10% Fetal Bovine Serum (FBS) purchased from Gemini Bioproducts. Induction of hepcidin was measured in sub-confluent cultures following replacement of the medium for 48 hours with serum-free EMEM or serum-free EMEM containing 10 ng/ml Bone Morphogenetic Protein 6 (BMP6) (R&D Systems).

### Enzyme-linked immunosorbent assay (ELISA) analysis for secreted Hepcidin

Hepcidin was measured in conditioned medium from HepG2 cells using an ELISA kit from Bachem according to the manufacturer’s protocol.

### Liquid chromatography (LC) separation

All samples were analyzed using a nanoACQUITY UPLC system (Waters Corporation, Milford, MA) coupled online to a TSQ Vantage triple quadrupole mass spectrometer (Thermo Scientific, San Jose, CA). Solvents used were 0.1% formic acid in water (mobile phase A) and 0.1% formic acid in 90% acetonitrile (mobile phase B). Peptide separations were performed at a mobile phase flow rate of 400 nL/min using an ACQUITY UPLC BEH 1.7 μm C18 column (100 μm i.d. ×10 cm), which was connected to a chemically etched 20 μm i.d. fused-silica emitter via a Valco stainless steel union. 0.5 µl sample was injected for LC-SRM using a binary gradient of 5–20% B in 35 min, 20–25% B in 10 min, 25–38% B in 8 min, 38–95% B in 1 min, and 95% B for 6 min for a total of 75 min.

### Selected reaction monitoring (SRM) assay configuration

Mass spectrometric detection was performed using TSQ Vantage triple quadrupole mass spectrometer (Thermo Scientific). The TSQ Vantage was operated in the same manner as previously described^17^. Scan width of 0.002 m/z and a dwell time of 75 ms were set for all SRM transitions. The synthesized fully alkylated hepcidin-25 peptides were further evaluated for peptide response and fragmentation pattern. Optimal collision energy (CE) values were achieved by direct infusion of the individual peptides with CE ramping as depicted in Table 1. Precursor ions monitored were 651.3 m/z (z = 5) for the endogenous hepcidin-25 and 652.8 m/z (z = 5) for the heavy standard. The relative intensity ratios among the four final selected transitions for the SRM assay were predefined by internal standard heavy peptides. Matrix interference for a given transition that fell into the mass width of Q1 and Q3 from co-eluting peptides was determined by deviation from the expected relative intensity ratios between the transitions. While four transitions were monitored, the best two transitions with no matrix interference were used to generate calibration curve and hepcidin-25 quantification in media and clinical plasma samples (Table 1).

**Table 1:** The optimal collision energies (CEs) and selected transitions for alkylated hepcidin-25 (light and heavy versions).

Compound name	Precursor ion^5+^	Product ion	Collision energy	Fragment
Optimized SRM method and selected target compounds.				
DTHFPI**C**IF**C**CG**CC**HRSK**C**GM**CC**KT, M 3251.259	651.259	501.209	22	[b4]^+^
	**651.259**	**756.943**	**12**	**[y17]** ^**3+**^
	**651.259**	**794.637**	**11**	**[y18]** ^**3+**^
	651.259	847.981	11	[y19]^3+^
DTHFPI**C**IF**CC**G**CC**HRSK**C**GM**CCK**T, M 3259.971	652.862	501.209	22	[b4]^+^
	**652.862**	**759.614**	**12**	**[y17]** ^**3+**^
	**652.862**	**797.309**	**11**	**[y18]** ^**3+**^
	652.862	850.652	11	[y19]^3+^

C = C[+57.0], K = K[^13^C_6_,^15^N_2_], Ions chosen as quantifiers in bold, the others were used as qualifiers.

### Data analysis

SRM data acquired on the TSQ Vantage were analyzed using Xcalibur 2.0.7 (Thermo Scientific). Peak detection and integration were determined based on two criteria: (1) same retention time; (2) approximately same relative SRM peak intensity ratios across multiple transitions between light peptide and heavy peptide standard. All data were manually inspected to ensure correct peak detection and accurate integration. Signal to noise ratio (S/N) was calculated by the peak apex intensity over the highest background noise in a retention time region of ±15 s for the target peptides. The background noise levels were conservatively estimated by visually inspecting chromatographic peak regions. The limit of detection (LOD) and limit of quantification (LOQ) were defined as the lowest concentration point at which the S/N of surrogate peptide was at least 3 and 10, respectively. For conservatively determining the LOQ values, in addition to the requirements of the S/N to equal or be above 10, two other criteria were applied: the coefficient of variation (CV) at the concentration point be less than 20%; surrogate peptide response over the protein concentration be within the linear dynamic range. The light to heavy (L/H) SRM peak area ratio was used to generate the calibration curve and assess reproducibility. Microsoft Excel 2010 was used for statistical analysis and calibration curve plotting. The RAW data from TSQ Vantage were loaded into Skyline software^18^ to create high resolution figures of extracted ion chromatograms (XIC) of multiple transitions monitored for hepcidin-25.

## Results and Discussion

### Alkylated hepcidin assay configuration

**Figure 1:**
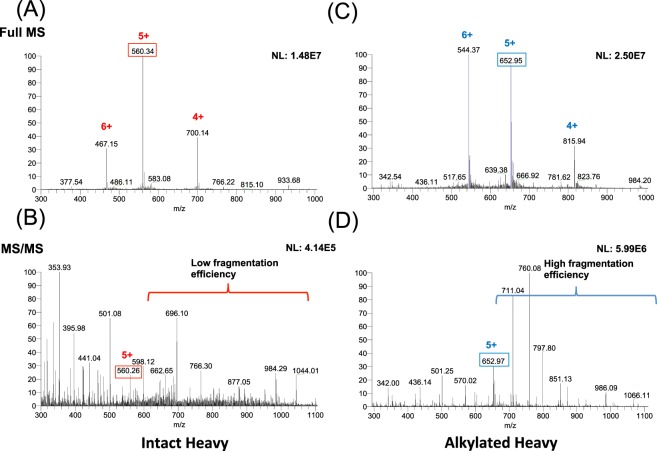
Optimization of SRM transitions of intact and alkylated isotopic hepcidin-25. MS spectra of intact (**A**) and alkylated isotopic hepcidin-25 (**C**). The most intense parent ions with the charge state of 5+, m/z 560.34 for intact isotope-labeled hepcidin-25 and m/z 652.95 for alkylated isotope-labeled hepcidin-25, were chosen to generate product ions. Representative MS/MS spectra that shows low fragmentation efficiency of the intact hepcidin (**B**) when compared to the alkylated form (**D**).

This work used the active hormone form of hepcidin to quantify hepcidin-25; DTHFPICIFCCGCCHRSKCGMCCKT. The mass spectrum for the intact (i.e. unalkylated) heavy form of hepcidin-25 is shown in Fig. 1. Figure 1A shows representative MS1 spectra for the intact (i.e. unalkylated) heavy hepcidin-25 and Fig. 1B shows representative MS2 spectra for dissociation of the intact heavy hepcidin-25. The MS2 spectra for the intact heavy hepcidin-25 is characterized by poor fragmentation as previously observed^19^. This is likely due to the four disulfide linkages which also makes synthesis of a consistent peptide product challenging^20,21^, and in part accounting for the significant variability reported for MS-based assays^14^. Thus, we reasoned that elimination of disulfide linkages via generation of an alkylated hepcidin-25 peptide product may significantly contribute to reducing the challenge associated with synthesis while simultaneously improving the low fragmentation efficiency associated with the disulfide linkages in intact hepcidin. Figure 1C,D show representative MS1 and MS2 spectra for the alkylated heavy form of hepcidin-25 respectively. Noticeably, the alkylated heavy form of hepcidin-25 provided a much higher fragmentation efficiency with a rich MS2 spectra (Fig. 1D) compared to the intact (i.e. unalkylated) heavy form of hepcidin-25 (Fig. 1B), which is consistent with our hypothesis. The list of fragment ions (b & y ions) for intact and alkylated heavy Hepcidin-25 are shown in Supplementary Table 4.

### Calibration curve for hepcidin-25 quantification in plasma

**Figure 2:**
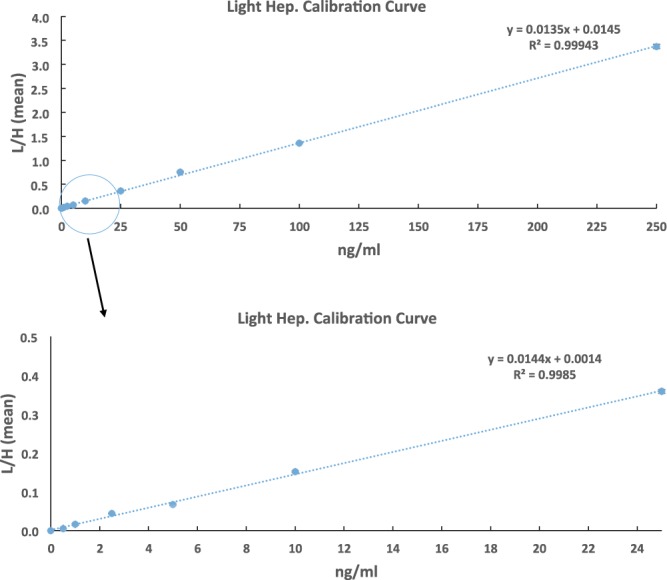
Calibration curve for hepcidin quantification. High-purity alkylated light hepcidin with a concentration range from 0.5–250 ng/mL was spiked into the control plasma with negligible endogenous hepcidin, with a fixed concentration (71.03 ng/mL) for alkylated heavy hepcidin.

To evaluate the hepcidin-25 assay performance a calibration curve was generated. High-purity alkylated light hepcidin-25 was spiked into non-cancerous human plasma with negligible endogenous hepcidin-25. We note that all clinical samples are treated with IAA to convert hepcidin to the analyzed alkylated hepcidin. The linearity was assessed by a 10-point calibration curve with concentrations ranging from 0.5 to 250 ng/ml (Supplementary Table 1). Figure 2 shows that the calibration curve has excellent linearity with a coefficient of correlation R^2^ ~ 0.99. Across the concentration range of the calibration curve, a median coefficient of variation (CV) of 2.66% was obtained with CV at 7.73% for the lowest concentration point (0.5 ng/mL) and at 2.36% for the highest concentration point (250 ng/mL) (Supplementary Table 1).

**Figure 3:**
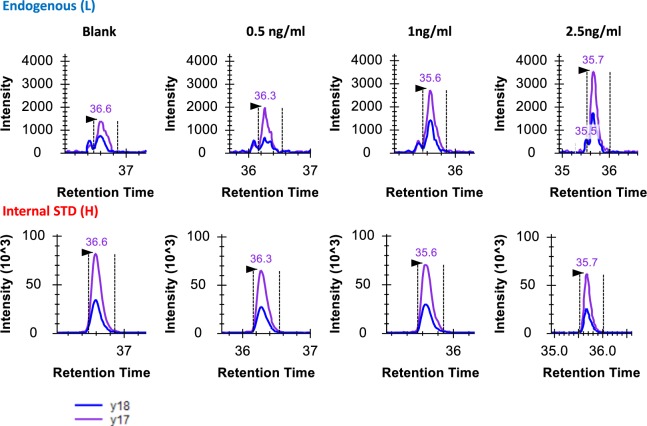
Extracted ion chromatograms (XICs) of transitions monitored for alkylated endogenous and heavy hepcidin at different spiked-in hepcidin concentrations. The black arrows indicated the location of SRM peak apex based on the retention time of heavy internal standards. Transition legend at bottom left hand of figure defines the blue and purple XIC traces for y18 and y17, respectively.

The calibration curve generated was leveraged to determine the LOQ and LOD for the hepcidin-25 assay developed when combined with the S/N ratio at each concentration point (Supplementary Table 1). Figure 3 depicts extracted ion chromatograms (XICs) of transitions monitored for alkylated endogenous and heavy hepcidin-25 at various concentrations. The LOD for the current assay was determined to be <0.5 ng/ml, and the LOQ was assessed to be 1 ng/ml. The LOD and LOQ values are better than what has been previously reported using intact hepcidin as an internal standard. For example, Delaby *et al*.^14^ recently reported a mass spectrometry-based hepcidin-25 assay with analytical validation performed according to ISO15189 norms. In that study they reported LOD of 2 ng/mL and LOQ of 6 ng/mL in serum. It is likely that the improved LOD and LOQ stem from the improved sensitivity offered by the higher fragmentation efficiency of the alkylated hepcidin when compared to the intact hepcidin bearing disulfide linkages.

### Endogenous hepcidin-25 quantification in human plasma

**Figure 4:**
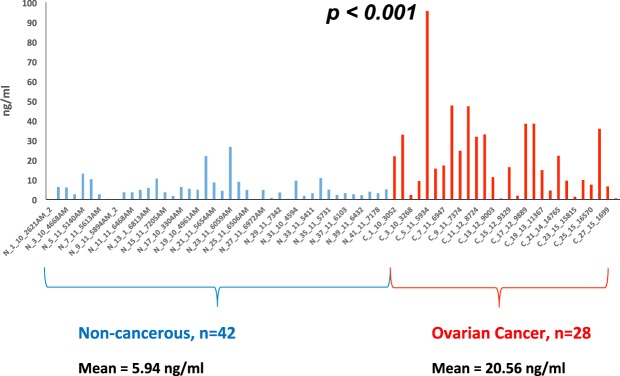
Application of developed hepcidin assay for quantification of endogenous hepcidin in clinical plasma samples. Cancer patients were found to have significantly higher hepcidin concentration than non-cancerous patients (p < 0.001).

Next, we applied our developed LC-SRM assay to quantify hepcidin concentration in human plasma specimens. Endogenous hepcidin-25 was measured in 70 clinical plasma samples (42 non-cancerous and 28 ovarian cancer patient samples). We note that all clinical samples are treated with IAA to convert hepcidin to the analyzed alkylated hepcidin. With the established calibration curve and the measured L/H peak area ratio for individual samples, the hepcidin-25 concentration in each sample could be calculated and expressed as ng/mL in plasma. The measured hepcidin-25 concentration ranged from 0 ng/mL to 95.57 ng/mL across non-cancerous and cancer samples. The measured hepcidin-25 concentrations are shown in Supplementary Table 1 and Fig. 4, they are largely within the range of our calibration curve. A prior study^14^ using a similar analytical approach to measure hepcidin-25 in serum indicated quantitation of hepcidin-25 in cohort samples did not exceed 140 ng/mL. That observation is largely consistent with the present study data where the highest hepcidin concentration reported in plasma was 95.6 ng/mL, in part supporting the validity of the assay developed using alkylated hepcidin-25 instead of traditional intact hepcidin-25.

Prior studies have suggested that hepcidin-25 serum levels may serve as a predictive biomarker in renal cell carcinoma and non-small cell lung cancer^22,23^. We also assessed the potential for hepcidin to discriminate between non-cancerous plasma and ovarian cancer patient plasma sample types. Non-cancerous plasma sample concentrations (n = 42) ranged between 0 ng/mL and 26.7 ng/mL with a median value of 4.28 ng/mL (a mean value of 5.9 ng/mL) (Supplementary Table 2). Ovarian cancer patient plasma concentrations (n = 28) ranged between 0.12 ng/mL and 95.6 ng/mL with a median value of 15.18 ng/mL (a mean value of 20.6 ng/mL). The increased hepcidin-25 amounts in serum of ovarian cancer patients relative to non-cancerous was statistically significant (p < 0.001) (Supplementary Table 2). Interestingly, this observation was consistent with prior reports of increased serum hepcidin-25 levels in renal cell carcinoma and non-small cell lung cancer^22,23^. Definitely, large clinical cohort studies are needed for the validation of hepcidin as a useful cancer biomarker.

### Quantification of hepcidin-25 concentration in spent media

**Figure 5:**
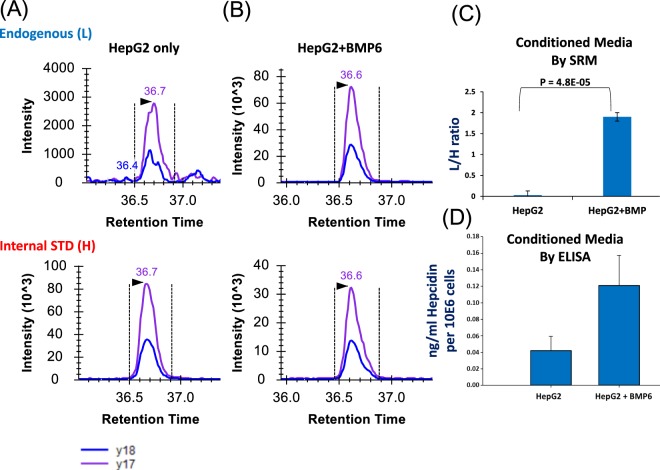
XICs of transitions monitored for alkylated endogenous and heavy hepcidin in hepatocytes cell media (HepG2) in the presence (**A**) and absence of BMP6 (10 ng/ml) (**B**).The black arrows indicated the location of SRM peak apex based on the retention time of heavy internal standard. Transition legend at bottom left hand of figure defines the blue and purple XIC traces for y18 and y17, respectively. (**C**) L/H hepcidin ratios for both HepG2 control and with BMP6 obtained from triplicate analysis with the standard error of mean. (**D**) Secretion of hepcidin in conditioned media. Secreted hepcidin in conditioned media was measured using ELISA. Hepcidin quantity was normalized to 1 × 10^6^ cells.

While the measurement of hepcidin in biological fluids (e.g., urine and plasma) is important in the context of the diagnosis and management of diseases in which iron metabolism is affected, the ability to measure hepcidin-25 in *in vitro* systems (e.g., cell culture) is also equally important to dissect and understand mechanisms underlying diseases in which iron metabolism is affected to identify novel therapies. Hepcidin in the context of cell culture is primarily partitioned into the condition media as opposed to being retained intracellularly. Therefore, we also developed an analytical approach for quantification of hepcidin-25 in spent media from mammalian cell culture systems. Specifically, we examined hepcidin-25 quantification in spent media from the hepatoma cell line HepG2. Because several studies have shown that bone morphogenetic proteins (BMPs) including BMP 2, 4, 6, and 9 stimulate hepcidin expression^24–26^, we also examined hepcidin-25 quantification in spent media from the hepatoma cell line HepG2 treated with BMP 6. Representative XICs of transitions monitored for alkylated endogenous and heavy hepcidin-25 in media from control and BMP 6-treated HepG2 cells are shown in Fig. 5A,B. As expected, BMP6 increased the abundance of hepcidin in conditioned media from HepG2 cells compared to untreated cells (Fig. 5C). Triplicate analysis showed an increase in hepcidin-25 after BMP6 stimulation (Fig. 5C and Supplementary Table 3). This was consistent with observations from immunoassay (ELISA) measurement of hepcidin under the same conditions (Fig. 5D). Thus, ELISA assay provides an orthogonal validation of hepcidin SRM assay.

## Conclusion

Hepcidin is a cysteine-rich tightly folded 25-residue peptide hormone containing four disulfide bonds, making endogenous hepcidin both challenging to synthesize^20,21^ and hampering reliable detection. Here we have developed a simple reliable targeted LC-SRM assay for quantification of hepcidin-25 in complex biological matrices by replacement of intact (disulfide linked) hepcidin standard with fully alkylated hepcidin standard. Our approach overcomes: (i) the challenges in synthesis and cost of generating intact hepcidin peptide, and (ii) the low fragmentation efficiency that characterizes the intact hepcidin peptide which challenges quantification accuracy. The new assay provided reliable detection of endogenous hepcidin-25 in human plasma samples and conditioned cell culture media. Additionally, our study suggests the potential for hepcidin to discriminate between non-cancerous plasma and plasma from patients with ovarian cancer. Larger cohort studies are definitely required to further validate hepcidin as a potential biomarker for ovarian cancer. In summary, we anticipate our newly developed assay will facilitate more reliable, sensitive, and low-cost quantification of hepcidin in complex biological samples.

## Supplementary information

Supplementary Information

Dataset 1

## Data Availability

All the Skyline-processed SRM results reported in this study can be accessed at Panorama (Access link: https://panoramaweb.org/Ixvl9u.url; the reviewer account: Email: panorama + pnnl5@proteinms.net, Password: W$VbZ2&R).
