# Supplementary material for: A Targeted Mass Spectrometric Assay for Reliable Sensitive Hepcidin Quantification
Source: Sci Rep. 2019 May 13;9:7264. doi: 10.1038/s41598-019-43756-9 (PMC6513854; doi:10.1038/s41598-019-43756-9)

Submitted to *Scientific Reports*

**A Targeted Mass Spectrometric Assay for Reliable Sensitive Hepcidin Quantification**

Ahmed Moghieb^1^, Lia Tesfay^2^, Song Nie^1^, Marina Gritsenko^1^, Thomas L. Fillmore^1^, Jon M. Jacobs^1^, Richard D. Smith^1^, Frank M. Torti^2^, Suzy Torti^2^, Tujin Shi^1¶^ and Charles Ansong^1¶^

^1^ Biological Science Division, Pacific Northwest National Laboratory, Richland, WA

^2^ Departments of Molecular Biology and Biophysics, University of Connecticut, Farmington, CT

**Running title:** Reliable sensitive quantification of hepcidin by mass spectrometry

**Keywords:** Targeted proteomics, hepcidin-25, quantifications, mass spectrometry

**¶To whom correspondence should be addressed:**

Dr. Charles Ansong

Integrative Omics Group

Biological Sciences Division

Pacific Northwest National Laboratory

Richland, WA 99352

**Tel:** (509)3716572

**Email:** [charles.ansong@pnnl.gov](mailto:charles.ansong@pnnl.gov)

Dr. Tujin Shi

Integrative Omics Group

Biological Sciences Division

Pacific Northwest National Laboratory

Richland, WA 99352

**Tel:** (509)3716579

**Email:** [tujin.shi@pnnl.gov](mailto:tujin.shi@pnnl.gov)

**SUPPLEMENTARY FIGURE 1:**

Sample preparation workflow for hepcidin-25 quantification.


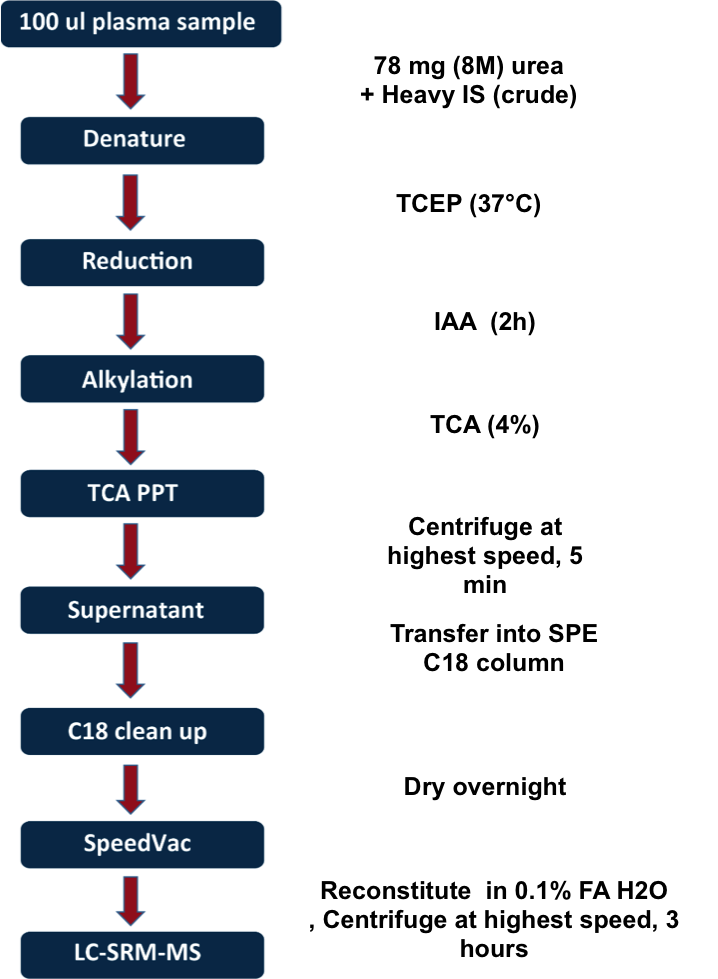

Supplement: Supplementary file 1 — Supplementary Information [file 41598_2019_43756_MOESM1_ESM.docx]
